# A Review of Synthetic Bone Grafts in Lumbar Interbody Fusion

**DOI:** 10.3390/bioengineering13030262

**Published:** 2026-02-25

**Authors:** Jaden Wise, Isabella Merem, Dahlia Wrubluski, Xuanzong Zhang, Ridge Weston, Min Shi, Maohua Lin, Frank D. Vrionis

**Affiliations:** 1 College of Medicine, Florida Atlantic University, Boca Raton, FL 33431, USA; 2 Department of Biomedical Engineering, Florida Atlantic University, Boca Raton, FL 33431, USA; 3 School of Computing and Informatics, University of Louisiana at Lafayette, Lafayette, LA 70504, USA; 4 Department of Neurosurgery, Marcus Neuroscience Institute, Boca Raton Regional Hospital, Boca Raton, FL 33486, USA

**Keywords:** synthetic bone graft substitutes, lumbar interbody fusion, osteoconductive scaffolds, graft–endplate interface, cage subsidence, early mechanical events, materials classification, outcome reporting variability

## Abstract

Lumbar interbody fusion is widely performed for degenerative, deformity-related, and instability-associated spinal conditions. Yet, reported outcomes remain variable across grafting strategies and surgical techniques. While advances in instrumentation and cage design improve immediate construct stability, successful arthrodesis depends on early graft behavior within the interbody environment. This includes positional stability, interface contact, and load transfer prior to mature bone formation. Synthetic bone graft substitutes are commonly used to supplement or replace autograft. However, the clinical literature describing these materials is heterogeneous with respect to composition, structural presentation, surgical context, and outcome reporting. This narrative review synthesizes clinical, translational, and biomechanical studies published between 2019 and 2025 that evaluate synthetic bone graft substitutes used in adult lumbar interbody fusion. Rather than comparing individual products or reported fusion rates, grafts are organized by material class and examined through early mechanical events such as graft migration, loss of graft–endplate contact, and cage subsidence. Across recent studies, variability in fusion definitions, imaging modalities, postoperative timepoints, and documentation of early mechanical events limits direct comparison and quantitative synthesis. These findings highlight the need for improved reporting consistency and greater emphasis on engineering-relevant variables in future investigations.

## 1. Introduction

Lumbar interbody fusion remains a commonly performed surgical procedure for degenerative, deformity-related, and instability-associated spinal conditions. Although advances in instrumentation and interbody cage design have improved immediate construct stability, fusion outcomes continue to vary across patient populations and surgical techniques [1]. In many cases, complications arise before radiographic evidence of osseous bridging is established, suggesting that early mechanical conditions at the graft site play a meaningful role in determining longer-term outcomes [2].

Synthetic bone graft substitutes are widely used in lumbar fusion to reduce donor-site morbidity associated with autograft harvesting and to provide consistent, scalable alternatives to allograft. However, published studies evaluating these materials report substantial variability in composition, architecture, handling characteristics, and clinical endpoints. Recent reviews have emphasized that differences in surgical approach, use of biologic adjuncts, imaging confirmation methods, and follow-up duration limit direct comparison across studies and complicate interpretation of reported fusion and complication rates [1,2].

Several failure mechanisms relevant to fusion success occur during the early postoperative period, when graft material is exposed to irrigation, packing forces, and nonuniform mechanical loading. Graft migration has been reported in lumbar fusion contexts, including posterior and interbody procedures [3]. In addition, endoscopic lumbar interbody fusion techniques are performed in irrigation-rich surgical environments, which has prompted discussion regarding graft handling and positional stability under such conditions [4]. Biomechanical investigations further demonstrate that inadequate graft–endplate contact and unfavorable load transfer contribute to endplate damage and cage subsidence, which may compromise segmental stability and the local environment for bone formation [5,6]. These events are often described descriptively rather than quantified using standardized definitions, further limiting synthesis across studies.

While these outcomes remain important, prior reviews have provided limited synthesis of early graft behavior within the implanted mechanical environment. In this phase, graft function is governed by containment within the cage, cohesion during packing, preservation of graft–endplate contact, and load transfer across the construct. These factors determine whether the graft maintains a stable interface long enough for incorporation [7].

Accordingly, this review synthesizes graft performance through operative, biomaterials, and engineering lenses, linking intraoperative handling and positional stability to scaffold architecture, degradation behavior, and implant–graft–endplate mechanics. Properties such as cohesion, resistance to displacement, volume stability, and interface mechanics may influence early graft performance. Yet, these factors are inconsistently reported in the clinical literature.

The objective of this narrative review is to synthesize recent literature published between 2019 and 2025 examining synthetic bone graft substitutes used in adult lumbar interbody fusion. Literature from this timeframe was selected to reflect contemporary interbody fusion workflows and reporting practices. These include expanded adoption of porous and additively manufactured cages, greater use of moldable composite grafts, and growth of minimally invasive and endoscopic techniques in which irrigation exposure may influence graft containment [1,4,7]. The review focuses on material design classes and their relationship to early mechanical failure modes, including migration, loss of graft–endplate contact, and subsidence-associated instability. Rather than advancing product-specific conclusions, this work aims to identify recurring patterns, highlight limitations in current reporting practices, and outline areas where additional mechanistic and comparative research is needed.

## 2. Methods

### 2.1. Review Design and Methodological Framework

This study was conducted as a structured narrative review integrating clinical, translational, and biomechanical literature relevant to synthetic bone graft substitutes used in adult lumbar interbody fusion. A narrative approach was selected because the available evidence is heterogeneous with respect to study design and graft composition. Additional variability exists in surgical technique, outcome definitions, and follow-up duration. This limits the validity of quantitative aggregation. The review structure followed established guidance for narrative synthesis in biomedical research, emphasizing transparency of search methods and critical appraisal of reporting practices rather than pooled effect estimates [8,9,10,11].

Narrative synthesis was considered appropriate given the limited availability of randomized comparative trials and the frequent use of mixed graft constructs in spine surgery research. Similar approaches have been recommended when evidence spans clinical cohorts, preclinical models, and biomechanical analyses that cannot be evaluated within a single statistical framework [12,13].

During synthesis, clinical studies were used to frame outcome reporting and complication relevance, while preclinical and computational investigations were used to interpret plausible mechanical mechanisms under defined boundary conditions. When interpretive discrepancies arose, clinical outcome data were prioritized, with preclinical and computational findings used to contextualize rather than supersede observed clinical patterns.

### 2.2. Literature Search Strategy

A structured literature search was conducted to identify clinical, translational, and biomechanical studies evaluating synthetic bone graft substitutes used in adult lumbar interbody fusion. Database selection was designed to capture both indexed biomedical literature and emerging translational work relevant to interbody fusion biomechanics and graft performance, particularly in studies evaluating early mechanical outcomes and graft–endplate interface behavior. PubMed served as the primary biomedical database, while Google Scholar and Open Evidence were included to identify conference abstracts, early online publications, and translational investigations not yet consistently indexed. Embase and Web of Science were not incorporated, as this review was designed as a structured narrative synthesis rather than a systematic review, and preliminary scoping demonstrated substantial overlap in indexed spine fusion literature across databases. Searches were limited to studies published between 1 January 2019 and 31 December 2025, and all databases were last queried in December 2025. Filters applied included English language and human subjects where applicable.

The PubMed database was queried using a combination of controlled vocabulary (MeSH terms) and free-text terminology combined with Boolean operators. Search terminology combined procedural language, graft material classifications, and mechanical outcome descriptors, with emphasis placed on studies evaluating graft containment, migration, subsidence, and early interface stability. The full PubMed search string was as follows: (“lumbar interbody fusion” [Title/Abstract] OR TLIF OR PLIF OR ALIF OR LLIF) AND (“synthetic bone graft” OR “bone graft substitute” OR “calcium phosphate” OR “bioactive glass” OR “ceramic graft” OR “biphasic calcium phosphate” OR “beta-tricalcium phosphate”) AND (“subsidence” OR “migration” OR “graft displacement” OR “endplate injury” OR “fusion rate” OR “interface mechanics”).

Google Scholar was queried using parallel keyword combinations reflecting procedural terminology, graft material classifications, and mechanical outcome descriptors. Representative searches included combinations of “lumbar interbody fusion,” “synthetic bone graft,” “calcium phosphate,” and “bioactive glass,” paired with outcome terms such as “subsidence,” “migration,” and “fusion rate.” Results were screened by relevance, with the first 200 records reviewed per query. Conference abstracts, early online publications, and translational investigations not yet indexed in PubMed were considered where clinically or mechanically informative. Open Evidence was queried using structured keyword searches aligned with PubMed terminology, incorporating procedural terms, graft material descriptors, and mechanical outcome language, with screening performed based on translational relevance to interbody fusion biomechanics. Reference lists of relevant systematic and narrative reviews were also manually screened to identify additional eligible studies not captured through database searches.

### 2.3. Eligibility Criteria and Study Selection

Studies were eligible for inclusion if they involved adult patients undergoing lumbar interbody fusion and evaluated synthetic bone graft substitutes either alone or as part of composite graft strategies. Eligible outcomes included radiographic fusion assessment, early mechanical events, or structural complications relevant to graft or cage performance. Randomized controlled trials, cohort studies, case series, registry analyses, and systematic reviews were included.

Preclinical and biomechanical studies were included when they addressed graft behavior, cage–endplate interaction, or interface mechanics with clear translational relevance. Studies focusing exclusively on cervical fusion, pediatric populations, infection management, or non-spinal applications were excluded. Selection decisions followed established principles for integrating heterogeneous evidence in spine surgery research [14].

### 2.4. Data Extraction and Synthesis Approach

Data extraction focused on study design, surgical approach, graft material class, cage characteristics, adjunct use, imaging modality, fusion definition, follow-up duration, and reported complications. Early mechanical events, including graft displacement, graft–endplate contact loss, cage subsidence, and endplate injury, were recorded when described.

Given substantial heterogeneity in outcome definitions and reporting practices, results were synthesized qualitatively [15]. Quantitative pooling was avoided in accordance with published recommendations cautioning against meta-analysis when methodological variability limits interpretability [16,17]. Studies were categorized by material class and failure pathway to facilitate comparison without implying material superiority.

### 2.5. Assessment of Reporting Quality

Reporting quality was assessed qualitatively with attention to consistency of fusion definitions, clarity of imaging criteria, disclosure of graft composition, and description of handling and containment strategies. Particular attention was given to whether early mechanical events were reported as predefined outcomes or only as complications.

This approach aligns with broader recommendations for improving transparency and interpretability in surgical outcomes research [18,19].

## 3. Material Classes of Synthetic Bone Graft Substitutes

Synthetic bone graft substitutes used in lumbar interbody fusion are best interpreted according to material class and structural presentation, as these features strongly influence handling characteristics, positional stability, resorption behavior, and interaction with the graft–endplate interface. In the contemporary literature, “synthetic” materials most commonly refer to ceramic- or glass-based scaffolds used either as standalone fillers or as components of composite graft formulations [1,2,20]. Distinctions between classes are often blurred in clinical studies, particularly when synthetics are combined with local autograft, bone marrow aspirate, or biologic adjuncts, complicating efforts to attribute outcomes to material design alone [21]. Accordingly, the classification used here emphasizes dominant architectural and compositional strategies rather than implying direct comparability across studies. Examples of commercially named products within these classes include Affinos^®^ (Kuraray Co., Ltd., Tokyo, Japan), MagnetOs Easypack Putty™ (Kuros Biosciences AG, Schlieren, Switzerland), and BioSphere Putty (Orthofix Medical Inc., Lewisville, TX, USA), although many cohorts report material class without specifying product.

### 3.1. Calcium Phosphate Ceramics

Calcium phosphate ceramics remain the most frequently reported synthetic scaffold class in lumbar fusion. These materials are typically formulated as hydroxyapatite, beta-tricalcium phosphate, or biphasic calcium phosphate blends designed to balance early structural persistence with gradual remodeling [1]. Mechanically, porosity, grain size, and phase composition influence both mechanical integrity and resorption kinetics. This may affect volume stability during the early postoperative period [7,22,23,24].

Recent clinical studies describe the use of calcium phosphate ceramics across a range of lumbar fusion techniques, most commonly with CT-based fusion assessment at approximately 12 months [2,25,26]. However, interpretation of reported outcomes is limited by substantial heterogeneity in surgical approach, cage geometry, endplate preparation, and use of adjunctive graft materials. In addition, fusion definitions and imaging thresholds vary across studies, and early mechanical events such as graft displacement or subsidence are not consistently reported. As a result, available evidence supports feasibility and widespread adoption rather than definitive conclusions regarding comparative performance [21].

### 3.2. Surface-Modified and Ion-Substituted Calcium Phosphate Ceramics

A subset of recent work has focused on modifying calcium phosphate ceramics through surface engineering or ionic substitution, with the aim of influencing early biological interactions while preserving the underlying scaffold structure [27,28]. Common strategies include ionic substitution (e.g., silicon, magnesium, or strontium) and surface texturing approaches intended to influence protein adsorption, osteogenic signaling, and early scaffold–bone interface activity without altering bulk structural geometry. These materials are most often evaluated as part of composite graft formulations rather than as standalone constructs, and clinical reports span both interbody and posterolateral fusion contexts.

Although surface modification is frequently described at a material level, clinical studies rarely provide sufficient detail regarding scaffold architecture, carrier composition, or handling characteristics to permit meaningful comparison across cohorts [2]. Consequently, surface-modified ceramics are grouped with conventional calcium phosphate scaffolds in this review, reflecting limitations in clinical reporting rather than an assessment of relative efficacy [21].

Direct comparative studies evaluating substituted versus unsubstituted calcium phosphate formulations remain limited, and most clinical cohorts report mixed graft constructs rather than isolating substitution-specific performance effects. In this context, substitution-related effects on early interface stability remain difficult to isolate clinically, and any mechanistic inferences should be interpreted within mixed-construct reporting environments.

### 3.3. Bioactive Glass-Based Graft Materials

Bioactive glass represents a distinct synthetic graft class with dissolution behavior and surface reactivity that differ from calcium phosphate ceramics. In lumbar fusion, bioactive glass has been reported in both granular and putty-based formulations, often in combination with instrumentation and other graft materials [1,29,30,31,32]. Clinical evidence describing its use is heterogeneous, with studies differing in formulation, application method, and outcome reporting.

Two recurring challenges characterize this literature. First, the same material category may appear in multiple physical presentations, yet handling-related variables such as packing method and containment strategy are not consistently described. Second, studies frequently treat “bioactive glass” as a single category despite compositional differences, limiting interpretability unless material details are explicitly disclosed. As with calcium phosphate ceramics, reported outcomes should therefore be interpreted in the context of variable reporting standards and mixed graft strategies [20].

Bioactive glass encompasses multiple compositional subclasses, including silicate- and borate-based formulations. Since most clinical cohorts do not distinguish these formulations or report full compositional detail, these limitations should be interpreted as literature-level reporting constraints rather than subtype-specific performance concerns.

### 3.4. Calcium Sulfate-Based Materials and Composites

Calcium sulfate-based materials are less frequently reported in lumbar fusion literature but appear intermittently as standalone fillers or as components of composite graft formulations [1,33]. From a materials standpoint, calcium sulfate is characterized by relatively rapid dissolution, which has prompted discussion regarding volume stability and mechanical persistence during early healing [33,34,35]. Accelerated dissolution may reduce early particle continuity and internal packing resistance, potentially influencing containment stability and graft–endplate load transfer during the immediate postoperative period.

In spine-specific studies, calcium sulfate is most often incorporated into composite grafts, making it difficult to isolate its independent contribution to reported outcomes. As a result, calcium sulfate-containing materials are best considered in terms of their potential influence on early graft geometry and resorption behavior rather than as a uniform substitute category across fusion techniques [2].

### 3.5. Composite Putties, Carriers, and Cohesion-Focused Architectures

Across recent lumbar fusion literature, the clinically used “graft” is frequently a composite construct, in which mineral particles are combined with a carrier intended to improve handling, conformability, and packing. These carriers vary widely in composition and degradation behavior, and clinical studies do not consistently report carrier-specific properties or their influence on early positional stability [1,2,36,37].

Within this broader category, several commercially available grafts employ fiber-stabilized or cohesion-focused composite architectures intended to improve handling and positional stability; however, publicly available data describing material composition and comparative clinical performance remain limited (e.g., OsteoFlo^®^ HydroFiber™). In the absence of standardized reporting, outcomes associated with putty-based or moldable composites should be interpreted with attention to what is not described, including packing technique, exposure to irrigation, containment strategy, and early radiographic events that may precede fusion success or failure [2,4,21].

### 3.6. Demineralized Bone Matrix as a Frequent Composite Component

Although not synthetic in origin, demineralized bone matrix appears frequently in lumbar fusion because it is commonly combined with ceramics or used as a carrier-like matrix in composite graft strategies [38,39,40,41]. Recent reviews emphasize substantial variability in DBM processing, osteoinductive potential, and clinical evidence, as well as inconsistent reporting across studies [40,42].

For the purposes of this review, DBM is included here because many cohorts described as evaluating “synthetic graft substitutes” involve DBM–ceramic composites, making it difficult to isolate ceramic effects unless the DBM contribution is explicitly acknowledged [2,43,44,45]. Recognizing this overlap is necessary for accurate interpretation of reported outcomes and for identifying gaps in comparative evidence.

**Figure 1:**
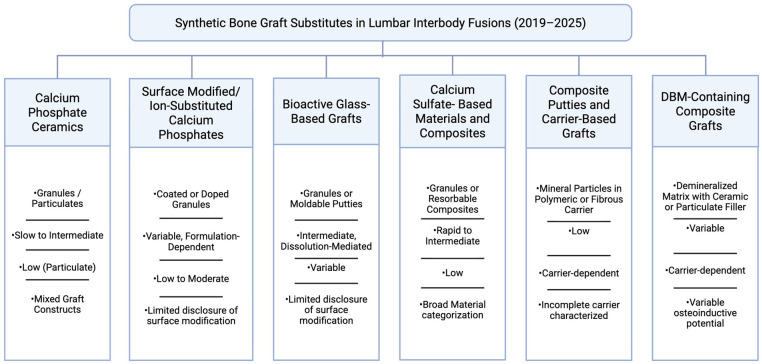
Material classes of synthetic bone graft substitutes used in lumbar interbody fusion and scaffold features influencing early graft stability.

Calcium phosphate ceramics generally demonstrate greater early structural persistence, while bioactive glass and calcium sulfate materials more often exhibit dissolution-driven changes in particle continuity and packing. Physical presentation also plays a role, as particulate grafts and moldable putties differ in cohesion, irrigation sensitivity, and containment behavior within the interbody space. These factors may influence early graft–endplate contact and displacement risk [21,46]. For clarity, synthetic graft substitutes are organized according to dominant material class and structural presentation, as summarized in Figure 1.

## 4. Early Mechanical Failure Modes at the Graft Site

**Figure 2:**
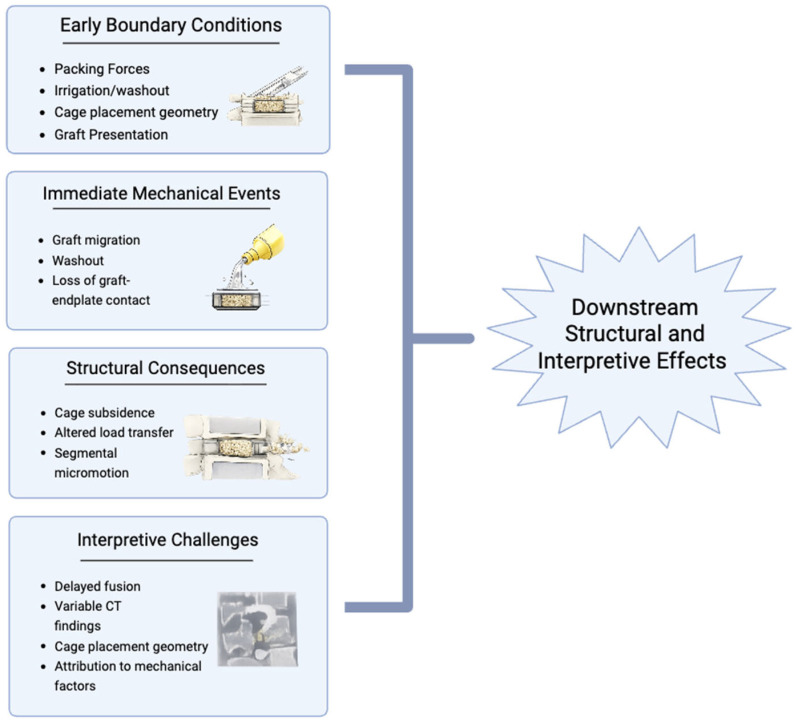
Early postoperative mechanical events at the graft site that disrupt conditions required for bridging bone formation.

Early postoperative outcomes after lumbar interbody fusion are influenced by mechanical events that occur before trabecular continuity is established across the motion segment. These events are variably defined and inconsistently reported in contemporary studies, complicating synthesis and emphasizing the need for clear, mechanism-based terminology [1,2,47]. Figure 2 summarizes the sequence of early mechanical events considered in this section, from intraoperative conditions to downstream structural consequences. Despite their potential clinical relevance, these early mechanical events are rarely studied as primary endpoints and are inconsistently defined across studies, limiting the ability to determine how early graft behavior influences fusion success.

### 4.1. Graft Displacement, Migration, and Washout

Displacement of graft material can occur intraoperatively or during the early postoperative period, particularly when graft is placed as particulates or small fragments without robust cohesion or containment [48,49]. Case reports and small series have documented anterior migration of graft material following posterior lumbar interbody fusion, demonstrating that even infrequent migration events can alter local graft geometry and, in some cases, place adjacent structures at risk [3,50,51]. Although the true incidence of clinically meaningful graft migration remains unclear, these reports highlight a fundamental mechanical consideration, loss of intended graft position alters the boundary conditions for fusion [52]. When present, however, displacement events may alter graft geometry, reduce effective fusion surface area, and in severe cases require revision.

From a clinical perspective, graft displacement represents more than a radiographic observation. Loss of containment may reduce scaffold contact with vascularized bone surfaces while also altering local load transfer across the interbody space. In this sense, early migration or washout may be interpreted as a biomechanical precursor to nonunion rather than an isolated technical complication.

In addition to migration after closure, graft displacement is discussed in technical literature describing biportal and endoscopic lumbar fusion techniques, where continuous saline irrigation is used throughout decompression and instrumentation. Several contemporary technique descriptions explicitly note concern for graft washout during insertion and describe mitigation strategies such as pausing irrigation and tamping during graft placement [4,53,54,55]. These reports do not establish a causal relationship between irrigation exposure and nonunion, but they support the narrower observation that washout is considered a plausible intraoperative risk under specific procedural conditions.

In clinical outcome studies, graft displacement and washout are rarely prespecified endpoints and are often reported only when associated with reoperation or overt complications. As a result, less severe or intraoperatively managed displacement events may be underrepresented in the literature, despite their potential relevance to early graft–endplate contact and subsequent fusion biology [2,56,57,58].

### 4.2. Loss of Graft–Endplate Contact and Void-Related Geometric Failure

Successful fusion requires maintenance of effective contact between graft material and host bone during the early healing period. Even when biologic potential is present, insufficient contact area, nonuniform packing, or early deformation of the graft can leave persistent voids or discontinuous interfaces that are mechanically unfavorable for bridging. Clinically, insufficient graft–endplate contact may delay or prevent osseous bridging, contributing to pseudarthrosis, persistent pain, or need for revision surgery [59].

Clinical evidence supports the relevance of early interface geometry. In posterior lumbar interbody fusion, insufficient contact between autograft and vertebral endplates has been associated with an increased risk of nonunion, linking early graft positioning to later radiographic outcomes [59]. More recently, clinical analysis in lateral lumbar interbody fusion supplemented with lateral plating identified insufficient endplate–bone graft contact as a risk factor for high-grade cage subsidence, further connecting early interface geometry to subsequent structural failure rather than treating subsidence as an isolated implant event [6,60].

Cage design can also influence graft–endplate relationships [61]. Comparative clinical work evaluating porous titanium cages with and without central graft windows reflects renewed interest in whether graft volume and continuity affect mechanical behavior and fusion assessment over time [62]. While such studies do not establish that any single cage architecture is universally preferable, they reinforce that graft behavior is inseparable from the geometric constraints imposed by the implant.

Loss of graft–endplate contact and void formation are likely underreported because they may not produce discrete early clinical symptoms. Their consequences may instead appear later as delayed fusion or ambiguous radiographic findings, particularly when early imaging and packing details are not described [2].

### 4.3. Endplate Injury and Cage Subsidence as Coupled Mechanical Events

Subsidence similarly extends beyond endplate violation alone. Reductions in interbody height may redistribute compressive forces, influence segmental alignment, and modify graft loading conditions during early healing. These changes alter the mechanical environment in which bridging bone formation occurs, linking subsidence to both structural and biologic outcomes [1]. From a clinical standpoint, subsidence may compromise foraminal height, contribute to recurrent neural compression, and has been associated in some series with delayed fusion or revision risk [63,64].

Cage subsidence is among the most frequently reported structural events following lumbar interbody fusion [65]. A systematic review comparing subsidence across fusion approaches highlights that subsidence occurs across techniques and that its incidence and clinical relevance depend on procedural context and patient factors [64,66].

Risk-factor analyses consistently identify endplate integrity and bone quality as major contributors [64]. Meta-analyses of oblique lumbar interbody fusion and transforaminal lumbar interbody fusion have reported associations between subsidence and older age, osteoporosis, endplate injury, and overdistraction [67,68,69,70,71,72]. These findings support an interface-mechanics perspective, in which subsidence reflects failure at the cage–endplate boundary under compressive loading, particularly when load is concentrated or supporting bone quality is reduced.

Subsidence may also alter the local graft environment. Changes in disc height and segmental alignment can reduce effective graft contact area and modify load transfer across the interbody space, potentially contributing to micromotion patterns that are unfavorable for bridging. However, subsidence is defined inconsistently across studies with respect to timing, imaging modality, and numeric thresholds, limiting synthesis even when subsidence is a prespecified outcome [2,47,65,73].

Biomechanical and computational studies further demonstrate that cage footprint, position, and endplate contact conditions influence stress distribution and subsidence risk, reinforcing the concept that early structural failure often reflects predictable interface mechanics rather than late biological failure alone [74,75,76]. The translational relevance of such models depends on accurate representation of material properties and boundary conditions consistent with clinical practice.

## 5. Synthesis of Recent Clinical and Translational Evidence

Across literature published between 2019 and 2025, eight clinical cohorts met inclusion criteria for structured extraction of synthetic bone graft substitute performance in adult lumbar interbody fusion. These studies span lateral, transforaminal, posterior, and mixed interbody approaches and reflect contemporary operative workflows incorporating porous titanium cages, moldable composite grafts, and minimally invasive or endoscopic techniques. Most cohorts evaluated synthetic grafts within mixed constructs rather than as isolated materials, frequently incorporating local autograft, DBM, or biologic adjuncts. As a result, interpretation of reported outcomes often reflects procedural context and implant environment as much as graft architecture alone. A structured summary of included studies and their reporting characteristics is presented in Table 1.

In addition to the eight clinical cohorts summarized in Table 1, the broader evidence base includes translational animal models, preclinical scaffold investigations, and computational simulations examining graft–implant–endplate mechanics under controlled boundary conditions. The majority of included cohorts evaluated graft performance in interbody fusion contexts, with fewer studies examining posterolateral or deformity-related applications.

**Table 1:** Clinical cohorts evaluating synthetic bone graft substitutes in adult lumbar interbody fusion published between 2019 and 2025.

Study	Fusion Approach	Graft Material Class	Adjuncts Used	Cage/Implant Context	Fusion Assessment	Follow-Up	Mechanical Events Reported
Biddau et al., 2024 [8]	ALIF	Mixed synthetic graft substitutes	±Autograft/DBM	Interbody cages	CT	12 months	Variably reported
Davis et al., 2025 [13]	TLIF	CaP composite putty	None (standalone)	Standalone interbody cage	CT	12 months	Low subsidence incidence reported
Hashimoto et al., 2023 [26]	PLIF	Synthetic graft fragments	None reported	Interbody cage	CT/imaging	Acute	Migration reported
Kumagai et al., 2019 [38]	LLIF	Porous β-TCP	None reported	Interbody cage	CT	12 months	Not reported
Nunley et al., 2024 [51]	Mixed interbody	Biphasic calcium phosphate	±Autograft/BMP-2	Interbody cages	CT + radiographs	12 months	Not reported
Wakelin et al., 2025 [75]	Lumbar interbody	Biphasic CaP (submicron surface)	Local autograft	Interbody cages	CT	12 months	Not reported
Westerlund & Borden, 2020 [77]	Lumbar interbody	Bioactive glass putty	±Autograft	Interbody cages	Radiographs ± CT	12 months	Not reported
Xie et al., 2023 [78]	Endoscopic LIF	Synthetic graft (NR)	None reported	Interbody cage	CT	Acute	Migration reported

### 5.1. Definitions of Fusion and Outcome Reporting

**Figure 3:**
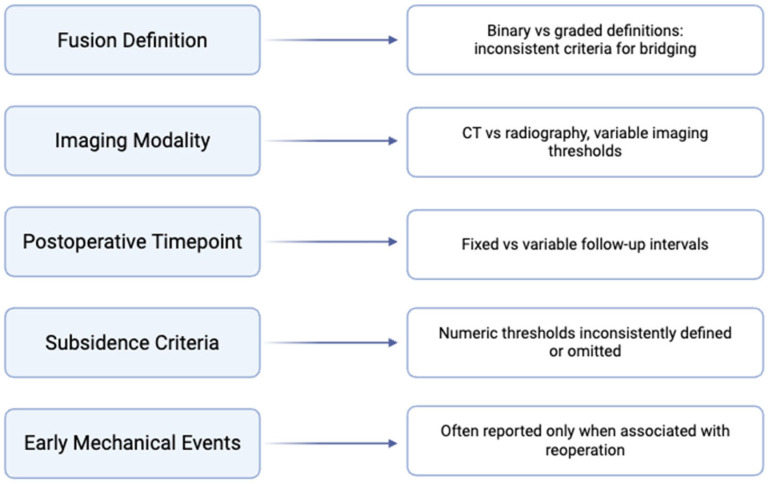
Variation in fusion definitions, imaging criteria, and subsidence reporting across contemporary lumbar interbody fusion studies.

Across contemporary lumbar interbody fusion studies, fusion is most commonly assessed using CT at a predefined postoperative interval, frequently around 12 months [77,79]. However, the criteria used to define bridging vary across cohorts, and thresholds for determining fusion are not applied uniformly [1,2,21,78]. Differences in patient selection, surgical approach, cage design, and use of adjunct graft materials further complicate interpretation when fusion outcomes are compared across studies. Variation in fusion definitions, imaging criteria, and reporting of early mechanical events across recent studies is summarized in Figure 3.

Several recent systematic reviews note that reported fusion rates often reflect heterogeneous study designs rather than intrinsic differences between graft materials [2,21,80]. In many cases, studies report fusion as a binary endpoint without detailing imaging criteria, early postoperative geometry, or handling conditions that may influence graft behavior [81]. As a result, synthesis of fusion outcomes across material classes is limited to broad feasibility rather than comparative inference.

The figure summarizes key methodological domains in which reporting varies across studies, including fusion definitions, imaging modality, timepoint specification, subsidence criteria, and documentation of early mechanical events. Variability across these domains limits direct comparison and supports qualitative synthesis.

### 5.2. Interpretation of Evidence by Material Class

Across included clinical cohorts, calcium phosphate-based scaffolds were most frequently represented, followed by composite putty formulations and bioactive glass-containing constructs, though most were evaluated within mixed graft environments rather than as isolated materials.

Evaluation of synthetic graft substitutes by material class is complicated by the fact that most clinical studies assess mixed constructs rather than isolated materials. Ceramic or glass-based scaffolds are frequently combined with local autograft, bone marrow aspirate, or demineralized bone matrix, and these combinations are often reported without sufficient detail to isolate the contribution of the synthetic component [1,21,43].

Where reported, commercially available graft products included biphasic calcium phosphate ceramics, fiber-reinforced composite putties, and bioactive glass formulations; however, product-level identification was inconsistently disclosed across cohorts, limiting comparative interpretation. Direct comparative clinical studies evaluating substituted versus unsubstituted calcium phosphate scaffolds remain limited, and most cohorts report mixed or composite graft constructs without isolating substitution-specific effects.

In studies reporting favorable fusion outcomes for calcium phosphate-based composites, variation in mixing practices, cage footprint, and surgical technique is common, limiting direct comparison across cohorts [25,26]. Trials and cohort studies evaluating calcium phosphate ceramics frequently include multiple fusion approaches or mixed indications, further reducing the ability to attribute outcomes to scaffold architecture alone [21,27]. These patterns support the interpretation that reported outcomes reflect procedural context as much as material class.

Bioactive glass-based graft materials are similarly represented across a range of formulations and clinical contexts. Reviews of this literature emphasize that differences in composition and presentation are often subsumed under a single category label, making broad conclusions difficult without detailed material disclosure [29,43]. While biologic plausibility and translational rationale are well described, clinical evidence remains constrained by inconsistent reporting.

### 5.3. Demineralized Bone Matrix in Composite Graft Strategies

Demineralized bone matrix appears frequently in studies described as evaluating synthetic graft substitutes, as it is commonly combined with ceramic scaffolds or used as a matrix in composite formulations [39,43]. Systematic review and meta-analytic evidence suggests that DBM–ceramic combinations can achieve fusion outcomes comparable to other grafting strategies across included spinal fusion studies, though interpretation is limited by heterogeneity in procedures and formulation-specific differences [39].

Clinical comparisons in interbody fusion contexts similarly reflect variability in definitions, imaging endpoints, and adjunct use, which limits generalization [2,41]. The frequent inclusion of DBM in synthetic graft cohorts highlights a recurring challenge in the literature: two studies may both be categorized as evaluating “synthetic” materials while differing substantially in osteoinductive contribution and handling behavior.

### 5.4. Early Mechanical Events and Downstream Interpretation

Early mechanical events are often underrepresented in clinical outcome reporting, despite their potential relevance to graft performance. Evidence linking insufficient early graft–endplate contact to later nonunion in posterior lumbar interbody fusion supports the view that early geometry influences radiographic outcomes [59]. More recent work identifying insufficient endplate–bone graft contact as a risk factor for high-grade cage subsidence after lateral lumbar interbody fusion further reinforces the connection between early interface conditions and structural failure [6].

Cage subsidence provides a parallel example of how early mechanical phenomena are inconsistently captured. A systematic review of lumbar fusion subsidence demonstrates that incidence and clinical relevance vary by approach and definition, and that thresholds for subsidence are not standardized across studies [65]. Meta-analyses in oblique and transforaminal lumbar interbody fusion consistently associate subsidence with age, bone quality, endplate injury, and overdistraction, supporting an interface-mechanics interpretation rather than a material-specific explanation [67,68]. Even when subsidence is included as an endpoint, variation in timing and measurement limits aggregation and comparison [2]. Across clinical and meta-analytic evidence, subsidence appears to be driven predominantly by implant positioning, endplate integrity, and host bone quality rather than graft material class alone.

Technique-specific cohorts further illustrate the influence of surgical context [82]. Studies in biportal endoscopic transforaminal lumbar interbody fusion report fusion and subsidence outcomes in relation to cage size and material within a single procedural framework, but these findings remain conditional on patient selection and workflow [83]. Technical discussions of irrigation-rich environments and graft handling similarly highlight that early mechanical boundary conditions differ across approaches, even when graft class labels are similar [4,53].

### 5.5. Translational Relevance of Preclinical and Computational Studies

Preclinical and computational studies contribute to understanding how cage footprint, position, and material stiffness influence interface stresses and stability. Their relevance to clinical interpretation depends on the extent to which boundary conditions and material properties reflect operative practice [7,45]. When these conditions are met, such models reinforce the concept that early mechanical behavior at the graft–implant–bone interface shapes downstream outcomes, even if clinical manifestation occurs later.

### 5.6. Summary of What Current Evidence Supports

Across studies published between 2019 and 2025, multiple synthetic graft material classes are used successfully in lumbar interbody fusion. However, the available evidence rarely supports strong comparative conclusions because outcomes are influenced by surgical technique, adjunct use, and reporting variability [1,2,21,43]. Across clinical and translational studies, a consistent observation is that early mechanical boundary conditions, including graft containment, graft–endplate contact, and interface load transfer, influence how fusion outcomes are interpreted. The structured extraction presented in Table 1 highlights the limited and heterogeneous reporting of early mechanical events across contemporary clinical cohorts.

Future studies would benefit from standardized definitions of fusion and subsidence, explicit reporting of graft presentation and mixing practices, and early imaging descriptors that capture interface integrity. Study designs that evaluate material classes under controlled surgical conditions may improve interpretability and help align engineering-relevant variables with clinically reported outcomes.

## 6. Conclusions and Future Directions

This review was conducted as a structured narrative synthesis and should be interpreted within that context. Study selection reflects database coverage and citation chaining rather than systematic pooling, and the available evidence base is weighted toward retrospective clinical cohorts alongside preclinical and biomechanical investigations. In addition, the present analysis intentionally focuses on early postoperative graft–implant mechanics rather than long-term implant survivorship or adjacent segment outcomes.

Outcomes following lumbar interbody fusion remain influenced by events that occur before mature osseous integration is established. Across recent literature, synthetic bone graft substitutes demonstrate performance that depends not only on biological potential but also on physical behavior within the interbody environment. Graft migration, loss of graft–endplate contact, and subsidence-associated instability are repeatedly described, yet these events are reported inconsistently and often without standardized definitions [1,2].

### Limitations

This review was conducted as a structured narrative synthesis and should be interpreted within that methodological context. Study selection reflects database coverage and citation chaining, with the evidence base weighted toward retrospective clinical cohorts alongside preclinical and biomechanical investigations. The analysis intentionally focuses on early postoperative graft–implant mechanics rather than long-term implant performance or adjacent segment outcomes. Accordingly, material-class interpretations are best viewed as mechanistic frameworks informed by available clinical reporting rather than definitive comparative efficacy comparisons.

Material design appears to influence how grafts behave during the early postoperative period, particularly with respect to cohesion, resistance to displacement, and volume retention. However, the current evidence base does not support strong comparative conclusions across material classes. Studies differ substantially in surgical technique, containment strategy, adjunct biologic use, imaging confirmation methods, and follow-up duration, making it difficult to isolate the contribution of material architecture from procedural and patient-specific factors [2].

Mechanical considerations are particularly relevant in contexts where graft materials are exposed to irrigation, packing forces, and nonuniform loading. Graft migration has been documented as an early complication in lumbar fusion, and endoscopic lumbar interbody fusion techniques introduce irrigation-rich environments that place additional emphasis on graft handling and positional stability [3,4]. Biomechanical studies further demonstrate that inadequate graft–endplate contact and unfavorable load transfer contribute to endplate damage and cage subsidence, which may compromise segmental stability and the local environment for bone formation [5,6].

Future work in this area would benefit from more consistent reporting of early mechanical outcomes. Standardized definitions for graft migration, displacement, and clinically meaningful subsidence thresholds would improve interpretability across studies. In parallel, translational and bench testing approaches that evaluate washout resistance, post-packing stability, and volume retention under physiologically relevant loading conditions could provide mechanistic context for observed clinical outcomes, particularly for techniques involving continuous irrigation.

Prospective comparative studies designed to evaluate material classes rather than individual products may help clarify how scaffold architecture influences early graft behavior. Such studies should aim to control for cage geometry, endplate preparation, and biologic adjuncts to reduce confounding. Computational modeling approaches may also contribute, provided that material properties and loading conditions are experimentally grounded and clinically relevant.

In summary, progress in synthetic bone graft design for lumbar fusion will depend on shifting emphasis from product-centered narratives toward materials-focused evaluation. Greater alignment between engineering-relevant metrics and clinically reported outcomes is needed to improve reproducibility and to better understand how early mechanical behavior influences fusion success.

## Data Availability

No new data were created or analyzed in this study.

## Abbreviations

The following abbreviations are used in this manuscript:

BMA	Bone marrow aspirtate
β-TCP	Beta-tricalcium phosphate
CT	Computed tomography
DBM	Demineralized bone matrix
LLIF	Lateral lumbar interbody fusion
OLIF	Oblique lumbar interbody fusion
PLIF	Posterior lumbar interbody fusion
TLIF	Transforaminal lumbar interbody fusion
